# Effects of RNA interference-mediated gene silencing of VEGF on the ultrafiltration failure in a rat model of peritoneal dialysis

**DOI:** 10.1042/BSR20170342

**Published:** 2017-08-31

**Authors:** Zhi-Kui Wang, Zhao-Xia Wang, Zhen-Ying Liu, Yue-Qin Ren, Zhong-Qi Zhou

**Affiliations:** Department of Nephrology, Linyi People’s Hospital, Linyi 276003, P.R. China

**Keywords:** Gene silencing, Peritoneal dialysis, Small interfering RNA, Ultrafiltration failure, Vascular endothelial growth factor

## Abstract

We investigated the effects of RNAi-mediated gene silencing of vascular endothelial growth factor (VEGF) on ultrafiltration failure (UFF) in rats with peritoneal dialysis (PD). Sprague–Dawley (SD) male rats were classified into normal, sham operation, and uremic model groups. Uremic rats were subcategorized into uremia, PD2, VEGF shRNA-2, vector-2, PD2 + Endostar, PD4, VEGF shRNA-4, Vector-4, and PD4 + Endostar groups. Peritoneal Equilibration Test (PET) was conducted to assess ultrafiltration volume (UFV) and mass transfer of glucose (MTG). mRNA and protein expressions of VEGF were detected using quantitative real-time PCR (qRT-PCR) and Western blotting. Immunohistochemistry was performed to detect microvessel density (MVD). Compared with the normal group, decreased UFV and increased MTG were observed in rest of the groups. Compared with the uremia group, UFV decreased, while MTG, expression of VEGFs, and number of new blood capillaries increased in the PD2, Vector-2, PD4, and Vector-4 groups. The PD4 and Vector-4 groups exhibited lower UFV and higher MTG than the PD2 group. In the VEGF shRNA-2, PD2 + Endostar, VEGF shRNA-4, and in PD4 + Endostar group increased UFV, reduced MTG and expression of VEGF, and decreased number of new blood capillaries were detected. Compared with the PD4 group, in the VEGF shRNA-4 and PD4 + Endostar groups, UFV increased, MTG and expression of VEGF decreased, and number of new blood capillaries reduced. VEGF expression was negatively correlated with UFV, but positively correlated with MTG. The results obtained in the study revealed that down-regulation of VEGF by RNAi could be a novel target approach for the treatment of UFF.

## Introduction

Ultrafiltration failure (UFF) can be understood as the durable loss of ultrafiltration capacity. This durable loss of capacity is reflected when the daily ultrafiltration volume (UFV) is less than 400 ml [1,2]. Common symptoms and signs of UFF include incisional hernia, exit site leak, hydrothorax, catheter malposition, scrotal swelling, and hemoperitoneum [3]. Generally speaking, there are four widely accepted causes of UFF: high effective peritoneal surface area, low osmotic conductance to glucose, low effective peritoneal surface area, and high total peritoneal fluid loss rate. The four main causative factors of UFF all have individual pathophysiological characteristics [4]. Peritoneal dialysis (PD) is a renal replacement therapy (RRT) for patients suffering from end-stage renal disease (ESRD) [5]. Globally, there are approximately 200000 patients currently suffering from end-stage renal failure and are in need of PD to sustain their life [6]. In addition, approximately 30% patients may suffer UFF following PD treatment for approximately 5 years [7]. UFF largely contributes to the technical failure seen in PD patients [8].

At present, there are few effective strategies available that could prevent UFF. Effective UFF prevention strategies include early prevention, treatment for peritonitis, maintenance of liquid, salt intake balance, and utilization of biocompatible solutions with lower levels of glucose degradation products (GDPs) in PD and angiogenesis [9]. However, many of the aforementioned therapies could not achieve the ideal outcome, thus searching for new and alternate therapies for the treatment of UFF is imminent.

Vascular endothelial growth factor (VEGF) has a great impact on assorted cells, such as neurones, skeletal muscle, and cardiac cells [10]. VEGF often functions with gene silencing, which could ultimately act to inhibit cell proliferation, migration, and invasion in relation to the treatment of various diseases [11]. Moreover, VEGF plays a significant role in wound healing, growth of certain solid tumors, and ascites formation [12]. Gene silencing refers to the regulation of gene expression and is effectively achieved by RNAi. siRNA molecules can be applied to help control the expression of specifically targetted genes [13]. This function is considered to be a powerful therapeutic method for the treatment of various diseases, including cancer, AIDS, liver and pulmonary diseases [14–17]. In the current study, we aimed to explore the effect of VEGF gene silencing on the UFF by using RNAi in a uremic rat model of PD, with the intention of detecting more effective methods for the prevention and treatment of UFF.

## Materials and methods

### Experimental animals

A total of 120 Sprague–Dawley (SD) male rats (aged: 6 weeks, weighing: 180–200 g), were purchased from Shanghai SLAC Laboratory Animal Co., Ltd. (Shanghai, China). All rats prior to the study were acclimated to a clean laboratory environment at 22–25°C, and achieved normal circadian rhythms with free access to food and water. All experimental procedures were conducted in accordance with the guidelines set by the International Association for the Study of Pain and also approved by the Laboratory Animal Ethics Committee of the Linyi People’s Hospital [18].

### The uremic rat model

In total, 120 SD male rats were randomly classified into normal (*n*=10; without any treatment), sham operation (*n*=10; treated with bilateral kidney capsulotomy), and uremic model groups (*n*=100; treated with 5/6 nephrectomy). The rats were anesthetized with 2% sodium pentobarbital (20 mg/kg, Sinopharm Chemical Reagent Co., Ltd., Shanghai, China) and fixed in the prone position. The skin, muscle, and fascia of the rats were successively opened at the junction 0.5 cm lateral to the spinal column and 1 cm below the left costarum in a consecutive fashion. Suturing followed the capsulotomy of the left kidney and removal of two-thirds of left kidney. All rats were intramuscularly injected with penicillin (North China Pharmaceutical Co., Ltd., Shijiazhuang, China) for 3 days in order to prevent infection. Next, the right kidney was removed using the same method, following 7 days’ recovery after the initial surgery. The nephrectomy on right kidney was performed by ligation of the renal pedicle at the right renal hilus. All rats were again intramuscularly injected with penicillin (North China Pharmaceutical Co., Ltd., Shijiazhuang, China) for a period of 3 days after surgery. All the rats were kept in a quiet environment. Blood acquired from the caudal artery of the rats was used to test renal function after 6 weeks. Blood from the caudal artery in rats was extracted before the sham operation and model establishment. Again, the blood was collected from caudal artery of rats for the assessment of renal function. The blood samples collected before sham operation and model establishment, and that of rats in the normal group were used as the normal reference values to determine whether the establishment of a uremic rat model had been successful or not. Rats with unsuccessful model establishment or dead were rejected.

### Construction of VEGF shRNA plasmid vector

According to the design principles of shRNA, the *VEGF* mRNA sequence of the rats was searched using the GenBank. The shRNA sense and antisense sequences of VEGF were designed using RNAi (Invitrogen Inc., Carlsbad, CA, U.S.A.). The TTCAAGAGA was selected as the loop structure in the shRNA template to avoid the formation of a termination signal (Table 1). The sequence alignment of the rat genome was completed using Blast software. The sequences were then synthesized by Shanghai GenePharma Co., Ltd. (Shanghai, China). DNA and oligo were dissolved in Tris-EDTA buffer (TE; pH 8.0) with a concentration of 100 μM. The corresponding shRNA sense and antisense sequences of VEGF were mixed with oligo solution. The annealing process was conducted in a PCR instrument in accordance with the following procedure: 95°C for 5 min, 85°C for 5 min, 75°C for 5 min, 70°C for 5 min, and finally reserved at 4°C. The shRNA template (10 μM) was obtained after annealing. BamHI and HindIII (Takara Shuzo Co. Ltd., Kyoto, Japan) restriction sites were inserted into both ends of the shRNA sequence template. The designed oligonucleotide sequence was linked to pSIREN-RetroQ-TetH vector (Takara Shuzo Co. Ltd., Kyoto, Japan). Next, the synthetic plasmid was transformed into *Escherichia coli* DH5α (TIANGEN Biotechnology Co. Ltd., Beijing, China) for the amplification of recombinant plasmid, which was incubated in LB medium containing ampicilin (Amp) overnight. The recombinant plasmid was extracted using a plasmid extraction kit (Beyotime Institute of Biotechnology, Shanghai, China) and sequenced by Invitrogen Inc. (Carlsbad, CA, U.S.A.) for identification of the validity of the basic sequence of shRNA fragment.

**Table 1:** shRNA sense and antisense sequences of VEGF

	Sequence
shRNA sense	5′-GATCCGGCCAGCACATAGGAGTTCAAGAGAAGATTCAAGACGTCTCTCCTATGTGCTGGCCTTTTTTGTCGACA-3′
shRNA antisense	3′-GCCGGTCGTGTATCCTCAAGTTCTCTTCTAAGTTCTGCAGAGAGGATACACGACCGGAAAAAACAGCTGTTCGA-5′

### Model of uremic rats undergoing PD

**Figure 1:**
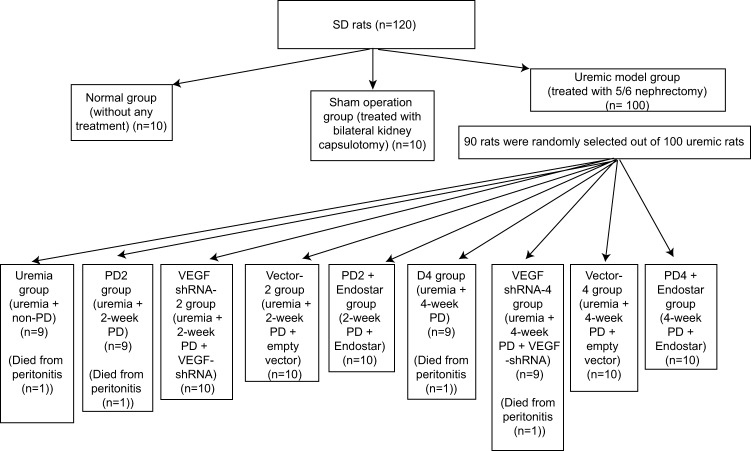
Flow diagram of experimental grouping and treatment

Uremic rats were randomly assigned into the uremia (uremia + non-PD), PD2 (uremia + 2-week PD), the VEGF shRNA-2 (uremia + 2-week PD + VEGF-shRNA), vector-2 (uremia + 2-week PD + empty vector), PD2 + Endostar (2-week PD + Endostar), PD4 (uremia + 4-week PD), VEGF shRNA-4 (uremia + 4-week PD + VEGF-shRNA), Vector-4 (uremia + 4-week PD + empty vector), and PD4 + Endostar groups (4-week PD + Endostar), with ten rats assigned to each group (Figure 1). With the exception of the uremia group, all the rats in the remaining eight groups underwent PD. The rats were anesthetized with 2% sodium pentobarbital (20 mg/kg, China Sinopharm Chemical Reagent Co., Ltd., Shanghai, China) and fixed at a prone position. The self-made catheter for PD (medical intravenous tubing with a heparin lock on one side and with many side holes on the other) was inserted 2 cm below the costarum. The end with holes was inserted into the abdominal cavity of rats. A subcutaneous tunnel was formed in unison with incision to the midpoint between the two ears of rats. Normal saline solution (20 ml) was administered via the catheter to check for any possibility of leakage. The catheter implantation was established successfully if the previously administered saline solution flowed out in a smooth manner. All rats were intramuscularly injected with penicillin to prevent infection, followed by injection with 50 U heparin (Beijing Science Sun, Inc., Beijing, China) for blockage of catheter. The rats were infused with 4.25% double dialysate (0.03 ml/g, Baxter International Inc., U.S.A.). During the period of the instillation of 4.25% double dialysate, rats in the VEGF shRNA-2 and VEGF shRNA-4 groups received intravenous injection of VEGF shRNA plasmid (1.5 nmol) for 14 and 28 days, respectively. The rats in the Vector-2 and Vector-4 groups were instilled with an empty pSilencer plasmid, while the other conditions were the same as the conditions of the VEGF shRNA group. During the period of the instillation of 4.25% double dialysate, rats in the PD2 + Endostar and PD4 + Endostar groups received subcutaneous injection of Endostar (40 mg/kg) once every 2 days [19], 7 and 14 times, respectively, until the 14th and 28th day of PD.

### Peritoneal Equilibration Test and renal function assessment

After 2 days of PD (specimens were selected from the uremia group and PD4 group at the same time), a 4-h Peritoneal Equilibration Test (PET) was performed to determine UFV and mass transfer of glucose (MTG) in rats in each group. Next, 4.25% double dialysate (25 ml) was infused into the rats, and 1 ml dialysate was reserved to detect UFV and MTG of the rats from the initial period at the beginning. A longitudinal incision was made in the abdominal wall following a 4-h period. Most of the fluid within the peritoneal cavity was extracted with a syringe and was subsequently measured. The remaining fluid in the peritoneal cavity was collected using gauze and then weighed. At the 4-h point, 2 ml dialysate was reserved to detect the UFV and MTG of rats. Before and after PD, a blood sample (2 ml) was extracted for renal function assessment detection of blood urea nitrogen (BUN) and serum creatinine (SCr). UFV (UF, ml) = the volume of fluid extracted from the peritoneal cavity with a syringe + (the weight of wet gauze – the weight of gauze before using) – 25 ml; MTG (nmol/kg) = (glucose concentration at the beginning of PD × the initial volume of injected dialysate) – (glucose concentration after PD × the final volume of reserved dialysate).

### Quantitative real-time PCR

Peritoneal tissue was obtained from the rats at the end of PET, prior to death. For the quantitative real-time PCR (qRT-PCR) analysis, total RNA was isolated from the peritoneal tissues using TRIzol reagent (Invitrogen Inc., Carlsbad, CA, U.S.A.). Nano Drop2000 (Thermo Fisher Scientific, Rockford, IL, U.S.A.) was applied to test the purity as well as the concentration of the RNA. The primers for qRT-PCR were designed using the Primer Premier version 5.0 software (Premier Biosoft International, Palo Alto, CA, U.S.A.), according to gene sequences published by GenBank database (Table 2). The primers were synthesized by Shanghai GenePharma Co., Ltd. (Shanghai, China). ABI PRISM 7500 real-time PCR System (ABI Company, Oyster Bay, NY) was used for PCR amplification. PCR was performed with 12.5 µl SYBR Premix Ex Taq II (Takara Biotechnology Co., Ltd., Dalian, China), 1 µl forward primer, 1 µl reverse primer, 8.5 µl sterile water, and 2 µl template. *β-actin* gene served as an internal reference. PCR reaction conditions: predenaturation for 3 min at 95°C, 40 cycles of denaturation for 30 s at 94°C, annealing for 30 s at 54°C, and extension for 1 min at 72°C. A solubility curve was drawn to evaluate the reliability of the results obtained from the PCR assessment. Relative expression of the target gene was calculated using the 2^−ΔΔ*C*^_t_ method. Formula: Δ*C*_T_ = *C*_T(target gene)_ − *C*_T(internal reference)_; ΔΔ*C*_t_ = Δ*C*_t(experimental group)_ − Δ*C*_t(control group)_ [20].

**Table 2:** Primer sequences of VEGF and β-actin for qRT-PCR

Gene	Primer sequence
*VEGF*	Forward: 5′-GCACCCATGGCAGAAGG-3′
	Reverse: 5′-CTCGATTGGATGGCAGTAGCT-3′
*β-actin*	Forward: 5′-CGCCCAGCACGATGAAA-3′
	Reverse: 5′-CCGCCGATCCACACAGA-3′

### Western blotting

Total protein was extracted from peritoneal tissues of rats. The concentration of protein was determined using the BCA Kit (Boster Bio-Engineering Co. Ltd., Wuhan, China). The protein samples were boiled with buffer solution at 95°C for 10 min, at a concentration of 40 µg/per well. The protein samples were separated by 10% SDS/PAGE (Boster Bio-Engineering Co. Ltd., Wuhan, China) with a voltage from spacer gel at 80 V to separation gel at 120 V and were then wet-transferred on to PVDF membranes with a constant voltage of 100 mV for 90–120 min. The membranes were sealed with 5% BSA at room temperature for 1 h, and then incubated with primary antibodies against VEGF (1:1000; Abcam Inc., Cambridge, MA, U.S.A.) and β-actin (1:1000; Abcam Inc., Cambridge, MA, U.S.A.) at 4°C overnight. After washing with TBS and Tween 20 (TBST) three times (5 min per wash), the membranes were incubated with the secondary antibody at room temperature for 1 h. Next, the membranes were washed three times with TBST (5 min per wash). The protein bands were visualized by chemiluminescence reagent. β-actin served as an internal reference indicator. The gray values of the protein bands were analyzed using ImageJ software.

### Immunohistochemistry

The peritoneal tissues of rats in each group was fixed with formalin and embedded with paraffin. The tissues were sliced into six to eight serial sections with 3 µm in thickness, placed on cation-coated slides, and then baked on a 65°C baker for 2 h. The sections were dewaxed with dimethylbenzene for 10 min, dehydrated by gradient ethanol, and then rinsed three times with distilled water (2 min per rinse). Next, the sections were immersed in TE buffer solution (pH 9.0; Boster Biological Technology Co., Ltd., Wuhan, China) for antigen retrieval under high pressure and rinsed with distilled water again. The sections were immersed in 3% H_2_O_2_, followed by incubation away from light at room temperature for 10 min, and rinsed with PBS (Boster Biological Technology Co., Ltd., Wuhan, China). The normal goat serum was added into the sections at 37°C for a 10-min incubation. The rabbit anti-rat CD34 primary antibody (1:100, Abcam Inc., Cambridge, MA, U.S.A.), diluted by PBS, was added to the sections for a 24-h incubation in refrigerator at 4°C. On the second day, after being rinsed with distilled water, the sections were incubated with the second antibody (rabbit anti-rat; Boster Biological Technology Co., Ltd., Wuhan, China) at 37°C for 30 min, and then rinsed with distilled water again. After being stained by diaminobenzidine (DAB), the sections were counterstained by Hematoxylin (Boster Biological Technology Co., Ltd., Wuhan, China) for 1–2 min. The sections were then differentiated by hydrochloric acid alcohol and washed using tap water. The differentiation of the sections was observed under a microscope. Sections were mounted in neutral gum after dehydration with ethanol.

The densest area of blood capillaries was visible under the microscope, and the number of blood capillaries was then counted. The vascular endothelial cells (VECs) were labeled with CD34 monoclonal antibody, and the number of stained microvessels was counted. The cytoplasm of the microvascular endothelium was dyed brown. A single staining vascular endothelial cell with distinct coloration and background or VEC cluster was considered as a microvessel. The number of microvessels was counted as follows: after the dense area of blood capillaries was found under the low-power field, the number of microvessels was then recorded under a high-power field [21,22]. Five high-power fields were randomly selected for each section to observe the dense area of blood capillaries. After counting, the mean value was regarded as the microvessel density (MVD).

### Statistical analysis

SPSS 18.0 statistical software (SPSS, Chicago, IL, U.S.A.) was utilized for the statistical analysis of the results of the study. Measurement data were presented as mean ± standard deviation (S.D.). A *t* test was applied to compare the measurement data in accordance with normal distribution between two groups. A one-way ANOVA was used for comparisons amongst multiple groups. Pearson correlation analysis methods were performed to explore the correlations of VEGF expression with UFV and MTG. Bilateral *P*<0.05 was regarded as being of statistical significance.

## Results

### Renal function of uremic rats before and after model establishment

The results indicated that there was no obvious difference in BUN and SCr of rats amongst the normal, sham operation, and uremia groups prior to model establishment (both *P*>0.05). BUN and SCr of rats in the uremia group were significantly increased in the sixth week of model establishment (both *P*<0.05), however no change was detected in the normal and the sham operation groups before model establishment (both *P*>0.05). At the sixth week after model establishment, when compared with the normal group, there were no differences in BUN and SCr of rats in the sham operation group (both *P*>0.05), while the BUN and SCr of rats displayed noticeable increase in the uremia group (both *P*<0.05). The results obtained indicated that the uremic rat models (*n*=100) had been successfully established (Table 3).

**Table 3:** Comparison of renal function of rats amongst the normal, sham operation, and uremia groups

Indicator	Normal group (*n*=10)	Sham operation group (*n*=10)	Uremia group (*n*=100)
Urea nitrogen (mmol/l)			
Before model establishment	2.46 ± 0.41	2.56 ± 0.25	2.43 ± 0.27
At the sixth week	2.66 ± 0.23	2.74 ± 0.39	8.81 ± 0.95^*†^
SCr (μmol/l)			
Before model establishment	23.53 ± 2.22	24.05 ± 3.12	30.03 ± 4.85
At the sixth week	25.81 ± 4.21	26.38 ± 3.89	73.01 ± 4.13^*†^

*P<0.05 compared with before model establishment; ^†^*P*<0.05 compared with the normal group.

### UFV and MTG of uremic rats undergoing PD in 11 groups

A total of 90 rats were randomly selected out of 100 uremic rats and divided into 9 groups, with 10 rats in each group. At the 28th day of PD, there was no record of death in rats amongst the normal and sham groups, however, there were four rats that died from peritonitis in the uremia, PD2, PD4, and VEGF shRNA-4 groups with one death in each group. No deaths were recorded in the other five groups. Thus, there was no difference in the survival rates of rats amongst groups in the study. Compared with the normal group, UFV and MTG of rats in the sham-operation group remained stable (all *P*>0.05), while UFV of rats significantly decreased, in addition to distinct MTG increased in the other seven groups (all *P*<0.05). Compared with the uremia group, UFV and MTG remained stable in the VEGF shRNA-2, PD2 + Endostar, VEGF shRNA-4, and PD4 + Endostar groups (*P*>0.05). In comparison with the uremia group, the UFV of rats exhibited significant decrease, while evident increase in MTG were seen in the PD2, PD4, and Vector-4 groups (all *P*<0.05). Compared with the PD2 group, the PD4 and Vector-4 groups exhibited distinct decrease in UFV while displaying increase in MTG (all *P*<0.05). The VEGF shRNA-2, PD2 + Endostar, VEGF shRNA-4, and PD4 + Endostar groups displayed significantly higher UFV and lower MTG level (all *P*<0.05). Compared with the PD4 group, there were no significant differences in UFV and MTG of rats in the Vector-4 group (*P*<0.05), while the UFV of rats was notably elevated and MTG reduced in the VEGF shRNA-4 and PD4 + Endostar groups (all *P*<0.05).

On the 28th day of PD, compared with the normal group, the weights of the rats remained stable in the sham operation, PD2, VEGF shRNA-2, Vector-2, PD2 + Endostar, PD4, VEGF shRNA-4, Vector-4, and PD4 + Endostar groups (all *P*>0.05). Moreover, the weight of rats evidently reduced in the uremia group (*P*<0.05). Compared with the uremia group, the weight of rats significantly increased in the PD2, VEGF shRNA-2, Vector-2, PD2 + Endostar, PD4, VEGF shRNA-4, Vector-4, and PD4 + Endostar groups (all *P*<0.05) (Table 4). Our results suggested that the uremic rat models of PD had been successfully established. This was largely due to the uremic rats with PD being exposed to high-glucose dialysate with non-biological compatibility for an extended period of time. Additionally, there was a loss of weight with reduced peritoneal ultrafiltration function. Therefore, VEGF gene silencing mediated by shRNA and Endostar treatment was concluded to alleviate UFF in the uremic rat model of PD.

**Table 4:** Comparison of UFV and MTG amongst the 11 groups (mean ± S.D., *n*=10)

Group	UFV (ml)	MTG (mmol/kg)	Weight	
			On the 1st day (g)	On the 28th day (g)
Normal group	9.97 ± 0.74	11.01 ± 0.56	339.23 ± 5.77	430.72 ± 7.28
Sham operation group	9.37 ± 0.96	10.71 ± 0.42	357.16 ± 6.42	427.33 ± 10.99
Uremia group	5.42 ± 0.51*	13.37 ± 1.05*	347.41 ± 5.19	343.85 ± 5.25*
PD2 group	2.91 ± 0.29*^†^	15.04 ± 0.97*^†^	359.04 ± 9.99	420.55 ± 7.91^†^
VEGF shRNA-2 group	5.83 ± 0.68*^‡§^	12.79 ± 0.27*^‡§^	344.32 ± 6.55	419.67 ± 8.57^†^
Vector-2 group	2.79 ± 0.24*^†§^	15.69 ± 0.38*^†§^	350.05 ± 7.66	422.38 ± 6.76^†^
PD2 + Endostar group	5.22 ± 0.28*^‡§^	13.04 ± 0.47*^‡§^	348.66 ± 5.99	424.77 ± 9.94^†^
PD4 group	1.14 ± 0.45*^†‡^	17.99 ± 0.84*^†‡^	357.72 ± 6.46	425.97 ± 9.82^†^
VEGF shRNA-4 group	4.89 ± 0.87*‡^§^	13.16 ± 0.93*‡^§^	351.61 ± 6.75	431.24 ± 7.21^†^
Vector-4 group	1.49 ± 0.12*^†‡^	18.06 ± 0.89*^†‡^	341.08 ± 7.24	421.61 ± 8.15^†^
PD4 + Endostar group	4.99 ± 0.39*^‡§^	13.06 ± 0.55*^‡§^	349.33 ± 8.65	427.63 ± 6.93^†^

*P<0.05 compared with the normal group; ^†^*P*<0.05 compared with the uremia group; ^‡^*P*<0.05 compared with the PD2 group; ^§^*P*<0.05 compared with the PD4 group.

### VEGF expression inhibited by shRNA and Endostar treatment

**Figure 2:**
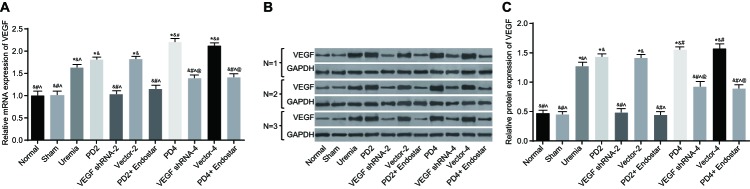
Comparison of mRNA and protein expression of VEGF amongst the 11 groups (**A**) Comparison of mRNA expression of VEGF detected by qRT-PCR amongst the 11 groups (*n*=10, and *n* (uremia, PD2, PD4, and VEGF shRNA-4 groups)=9); (**B**) band analysis of VEGF proteins detected by Western blotting in each group (repeated for three times; *n*=10, and *n* (uremia, PD2, PD4, and VEGF shRNA-4 groups)=9); (**C**) comparison of relative protein expression of VEGF amongst the 11 groups (*n*=10, *n* (uremia, PD2, PD4, and VEGF shRNA-4 groups)=9); *, *P*<0.05 compared with the normal group; ^&^, *P*<0.05 compared with the uremia group; ^#^, *P*<0.05 compared with the PD2 group; ^∧^, *P*<0.05 compared with the PD4 group; ^@^, *P*<0.05, compared with the VEGF shRNA-2 group.

The findings provided by qRT-PCR and Western blotting techniques indicated that, when compared with the normal group, the protein and mRNA expressions of VEGF increased in the peritoneal tissues of rats in the uremia, PD2, Vector-2, PD4, VEGF shRNA-4, Vector-4, and PD4 + Endostar groups (all *P*<0.05). No significant differences were found amongst the normal, sham operation, VEGF shRNA-2, and PD2 + Endostar groups (*P*>0.05). Compared with the uremia group, the protein and mRNA expressions of VEGF increased in the peritoneal tissues of rats in the PD2, Vector-2, PD4, and Vector-4 groups (all *P*<0.05), while significantly decreased in the VEGF shRNA-2, PD2 + Endostar, VEGF shRNA-4, and PD4 + Endostar groups (all *P*<0.05). The protein and mRNA expressions of VEGF in the peritoneal tissues of the rats showed no significant differences between the PD2 and Vector-2 groups (*P*>0.05). Compared with the PD2 group, the protein and mRNA expressions of VEGF evidently increased in the PD4 and Vector-4 groups, while markedly decreased in the VEGF shRNA-2, PD2 + Endostar, VEGF shRNA-4, and PD4 + Endostar groups (all *P*<0.05). Compared with the PD4 group, the protein and mRNA expressions of VEGF significantly decreased in peritoneal tissues of rats in the VEGF shRNA-4, and PD4 + Endostar groups (all *P*<0.05), while there were no obvious differences in the Vector-4 group (*P*>0.05). Compared with the VEGF shRNA-2 group, the protein and mRNA expressions of VEGF significantly elevated in the VEGF shRNA-4 and PD4 + Endostar groups (all *P*<0.05), while no significant difference was found in the PD2 + Endostar group (*P*>0.05) (Figure 2). The results indicated that the expression of VEGF increased in rats with PD, while up-regulated in connection with long-term PD and high-glucose dialysate. The shRNA and Endostar treatment was thus thought to have a significant effect in relation to decreasing the expression of VEGF.

### The number of new blood capillaries reduced by VEGF gene silencing

**Figure 3:**
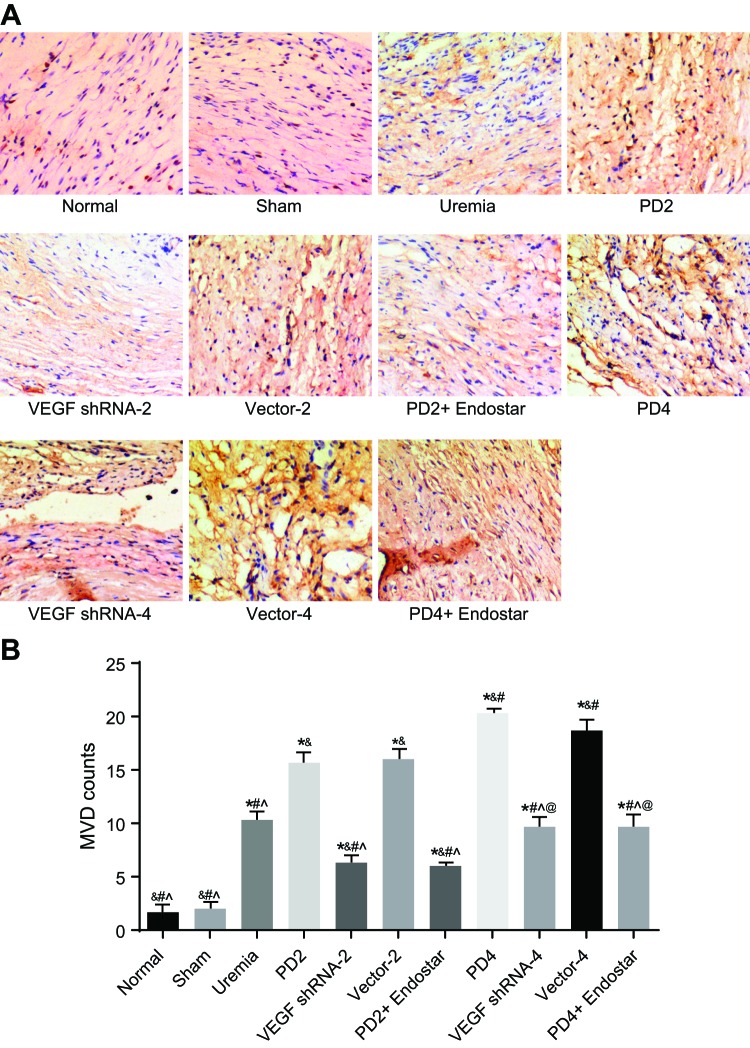
The number of new blood capillaries measured by immunohistochemistry in the 11 groups (*n*=3) (**A**) Images of stained new blood capillaries measured by immunohistochemistry; (**B**) comparison of the number of new blood capillaries amongst the 11 groups; *, *P*<0.05 compared with the normal group; ^&^, *P*<0.05 compared with the uremia group; ^#^, *P*<0.05 compared with the PD2 group; ^∧^, *P*<0.05 compared with the PD4 group; ^@^, *P*<0.05 compared with the VEGF shRNA-2 group.

Immunohistochemistry was performed to detect the expression of CD34 for the blood vessel counts in peritoneal tissues of rats, with three rats from each group. The results revealed that compared with normal group, the number of new blood capillaries significantly increased in the uremia, PD2, VEGF shRNA-2, Vector-2, PD2 + Endostar, PD4, VEGF shRNA-4, Vector-4, and PD4 + Endostar groups (all *P*<0.05). There were no obvious differences between the normal and sham operation groups (*P*>0.05). Compared with the uremia group, the number of new blood capillaries distinctly increased in the PD2, Vector-2, PD4, and Vector-4 groups (all *P*<0.05), while the VEGF shRNA-2 and PD2 + Endostar groups showed a significant reduction in the number of new blood capillaries (both *P*<0.05). The VEGF shRNA-4 and PD4 + Endostar groups displayed no noticeable difference in relation to the number of new blood capillaries, when compared with the uremia group (both *P*>0.05). No significant difference was observed in the number of new blood capillaries between the PD2 and Vector-2 groups (both *P*>0.05). Compared with the PD2 group, the PD4 and Vector-4 groups exhibited pronounced increase in the number of new blood capillaries (both *P*<0.05), while the VEGF shRNA-2, PD2 + Endostar, VEGF shRNA-4, and PD2 + Endostar groups all had significant decrease (all *P*<0.05). Compared with the PD4 group, the number of new blood capillaries in peritoneal tissues of rats markedly decreased in the VEGF shRNA-4 and PD4 + Endostar groups (both *P*<0.05). There was no significant difference in the number of new blood capillaries between the PD2 and Vector-4 groups (*P*>0.05). Compared with the VEGF shRNA-2 group, the number of new blood capillaries evidently increased in the VEGF shRNA-4 and PD4 + Endostar groups (both *P*<0.05), while the PD2 + Endostar group exhibited no evident difference (*P*>0.05) (Figure 3). The findings indicated that the number of new blood capillaries increased in PD rats with UFF. The number of new blood capillaries increased after long-term PD and high-glucose dialysate, while ultimately decreased by treatment with shRNA-mediated VEGF gene silencing and Endostar.

### Correlations of VEGF expression with UFV and MTG

**Figure 4:**
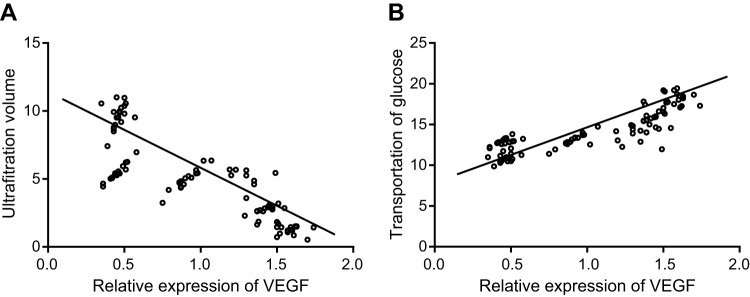
Pearson correlation analysis of correlations between VEGF expression, UFV, and MTG (*n*=106) (**A**) A significant negative correlation between UFV and VEGF expression; (**B**) a significant positive correlation between MTG and VEGF expression.

A Pearson correlation analysis was performed in order to explore the correlations of VEGF expression with UFV and MTG in peritoneal tissues of rats (*n*=110). The results indicated that there was a significantly negative correlation between UFV and VEGF expression (R = –0.814, *P*<0.001), while MTG was positively correlated with VEGF expression (R = 0.853, *P*<0.001) (Figure 4).

## Discussion

In order to reduce the loss of ultrafiltration capacity, the present study was conducted with the aim of investigating the effects of RNAi-mediated silencing of VEGF on the UFF in uremia rats with PD. The present study found that the down-regulation of VEGF, mediated by shRNA, can decrease the loss of ultrafiltration capacity in rats with PD.

Uremia syndrome can be defined as the terminal clinical manifestation of renal failure. The levels of BUN and SCr allow for the estimation of renal function [23,24]. Meanwhile, it has been reported that weight loss is a common symptom of uremia, and weight loss induced by bariatric surgery, physical exercise, or low-calorie diets is usually accompanied by a significant antiproteinuric effect [25]. Additionally, uremia has been reported to be associated with increased adipose tissue macrophage infiltration as well as concurrent muscle tissue mitochondrial dysfunction induced by inflammation and reactive oxygen species [26]. PD is an RRT method for patients with uremia. Peritonitis is currently one of the leading complications involved with PD, with approximately 18% of the infection-related mortality of PD patients caused by peritonitis [27,28]. In our experiments, the average levels of BUN and SCr of the rats presented a marked increase in the uremia group than in the normal group. Moreover, weights of the rats increased significantly in the PD2, VEGF shRNA-2, Vector-2, PD2 + Endostar, PD4, VEGF shRNA-4, Vector-4 and PD4 + Endostar groups, in comparison with the uremia group on the 28th day of PD. It has been reported that glucose absorption was one of the significant reasons for weight gain after PD [29], which was in accordance with the results provided from our study. UFF is regarded as a decrease in UFV [4]. High glucose, acting as a kind of the osmotic agent in PD solutions, triggers changes in the peritoneal membrane (PM) surface over time, which eventually causes PM deterioration, and consequently leads to the occurrence of UFF [5]. In our study, the results obtained indicated that when compared with the normal group, UFV decreased and MTG increased in the uremia, PD2, VEGF shRNA-2, Vector-2, PD2 + Endostar, PD4, VEGF shRNA-4, Vector-4, and PD4 + Endostar groups. The uremic rats with PD were exposed to high-glucose dialysate with non-biocompatibility for a long period of time, which caused a loss of ultrafiltration capacity. Compared with the PD4 group, the UFV elevated and MTG reduced in the VEGF shRNA-4 and PD4 + Endostar groups. VEGF gene silencing mediated by shRNA and Endostar treatment might alleviate UFF. VEGF is a heparin binding, dimeric glycoprotein that potently induces the proliferation and migration of endothelial cells [30]. VEGF is also considered as an essential element for the regulation of angiogenesis and vasculogenesis [31]. In addition, VEGF can promote angiogenesis, which may also consequently lead to UFF [1,32]. It has been demonstrated that siRNA, as a method for RNAi, presents an excellent gene silencing efficiency which can reduce *VEGF* mRNA and VEGF levels [17]. According to our study, the UFV of rats elevated and MTG reduced via the down-regulation of VEGF using another method for RNAi (shRNA), which thereby prevented UFF in PD rats.

In the current study, the results reported indicated that there was a significant negative correlation between UFV and VEGF expression, while MTG was positively correlated with VEGF expression. High-glucose PD fluid and uremic circumstance were highlighted as being related to the increasing expressions of VEGF in a PD time-dependent manner, possibly leading to UFF through angiogenesis [33]. Peritoneal angiogenesis can be inhibited after Endostar treatment by down-regulating the mRNA and protein expression of VEGF in uremic rats with PD, while VEGF secretion may be induced by high glucose [12]. Moreover, compared with the normal group, mRNA and protein expression of VEGF increased in the peritoneal tissues of rats in the uremia, PD2, Vector-2, PD4, VEGF shRNA-4, Vector-4, and PD4 + Endostar groups. No significant differences were found amongst the normal, sham operation, VEGF shRNA-2, and PD2 + Endostar groups. Thus, the findings indicated that expression of VEGF increased in rats with PD, while reduced in rats treated by shRNA against VEGF or Endostar.

The results of our study demonstrated that the number of new blood capillaries markedly increased in the PD2, Vector-2, PD4, and Vector-4 groups, while the VEGF shRNA-2 and PD2 + Endostar groups showed a significant reduction in comparison with the uremia group. In addition, the PD4 and Vector-4 groups displayed greater increase in the number of new blood capillaries than the PD2 group. Angiogenesis could be induced by VEGF family growth factors, such as VEGFR2 and VEGFR3, which play a critical role in the blood vessel growth [34]. Furthermore, VEGF served as a potential novel target for antiangiogenic and antilymphangiogenic therapies [35]. For example, the use of VEGF receptor inhibitor SU5416 from 46 to 65 hpf has been highlighted as being able to block the formation of the aortic arch (AA) blood vessels [36]. Our findings indicated that the number of new blood capillaries increased in PD rats with UFF. The number of new blood capillaries increased after long-term PD and high-glucose dialysate. Further, more decreases were observed after shRNA-mediated VEGF gene silencing as well as Endostar treatment. Hence, angiogenesis may be an important factor in the mechanism for the prevention of UFF in PD rats.

In conclusion, the results of the present study demonstrated that shRNA-mediated gene silencing of VEGF could potentially decrease the loss of ultrafiltration capacity by inhibiting VEGF expression in uremic rats with PD. However, the present study did not thoroughly investigate how shRNA VEGF affected expression of VEGF as well as the potential mechanism of shRNA-mediated gene silencing of VEGF in uremic rats undergoing PD. Moreover, more researches are necessary to seek an alternative and more effective strategy for the down-regulation of expression of VEGF.

## Abbreviations

BUN: blood urea nitrogen
MTG: mass transfer of glucose
PD: peritoneal dialysis
PET: peritoneal equilibration test
PM: peritoneal membrane
qRT-PCR: quantitative real-time PCR
RRT: renal replacement therapy
SCr: serum creatinine
SD: Sprague–Dawley
TBST: TBS and Tween 20
TE: Tris-EDTA
UFF: ultrafiltration failure
UFV: ultrafiltration volume
VEC: vascular endothelial cell
VEGF: vascular endothelial growth factor
