# Correction: Effects of RNA interference-mediated gene silencing of VEGF on the ultrafiltration failure in a rat model of peritoneal dialysis

**DOI:** 10.1042/BSR-20170342_COR

**Published:** 2020-05-14

**Keywords:** Ultrafiltration failure, Vascular endothelial growth factor, Small interfering RNA, Gene silencing, Peritoneal dialysis

The authors of the original article “Effects of RNA interference-mediated gene silencing of VEGF on the ultrafiltration failure in a rat model of peritoneal dialysis” (*Bioscience Reports* (2017) **37**, https://doi.org/10.1042/BSR20170342) have realised that the vector-4 group image of Figure 3 had been mistakenly placed into the PD4 group image of their immunohistochemistry experiment.

The correct Figure 3 is presented below.

**Figure 3:**
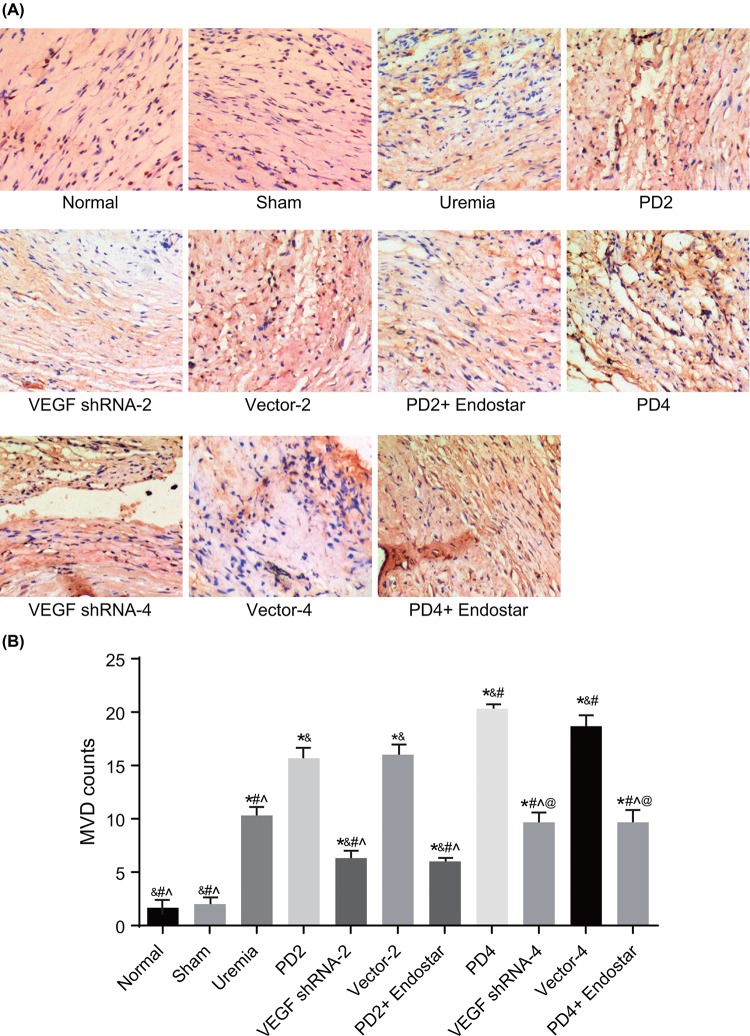
The number of new blood capillaries measured by immunohistochemistry in the 11 groups (n=3) (**A**) Images of stained new blood capillaries measured by immunohistochemistry; (**B**) comparison of the number of new blood capillaries amongst the 11 groups; *, P<0.05 compared with the normal group; ^&^, P<0.05 compared with the uremia group; ^#^, P<0.05 compared with the PD2 group; ^∧^, P<0.05 compared with the PD4 group; ^@^, P<0.05 compared with the VEGF shRNA-2 group.

The authors would like to express their apologies for any inconvenience caused to the readers of their published article by this error.

